# C1QTNF3 is Upregulated During Subcutaneous Adipose Tissue Remodeling and Stimulates Macrophage Chemotaxis and M1-Like Polarization

**DOI:** 10.3389/fimmu.2022.914956

**Published:** 2022-06-02

**Authors:** Peter Micallef, Milica Vujičić, Yanling Wu, Eduard Peris, Ying Wang, Belén Chanclón, Anders Ståhlberg, Susanna L. Cardell, Ingrid Wernstedt Asterholm

**Affiliations:** ^1^ Department of Physiology/Metabolic Physiology, Institute of Neuroscience and Physiology, The Sahlgrenska Academy at University of Gothenburg, Göteborg, Sweden; ^2^ Department of Microbiology and Immunology, Institute of Biomedicine, The Sahlgrenska Academy at University of Gothenburg, Göteborg, Sweden; ^3^ Sahlgrenska Center for Cancer Research, Department of Laboratory Medicine, Institute of Biomedicine, The Sahlgrenska Academy at University of Gothenburg, Göteborg, Sweden; ^4^ Wallenberg Centre for Molecular and Translational Medicine, University of Gothenburg, Göteborg, Sweden; ^5^ Department of Clinical Genetics and Genomics, Sahlgrenska University Hospital, Göteborg, Sweden

**Keywords:** adipose tissue, breast cancer, obesity, C1QTNF3, CTRP3, macrophage

## Abstract

The adipose tissue undergoes substantial tissue remodeling during weight gain-induced expansion as well as in response to the mechanical and immunological stresses from a growing tumor. We identified the C1q/TNF-related protein family member *C1qtnf3* as one of the most upregulated genes that encode secreted proteins in tumor-associated inguinal adipose tissue - especially in high fat diet-induced obese mice that displayed 3-fold larger tumors than their lean controls. Interestingly, inguinal adipose tissue *C1qtnf3* was co-regulated with several macrophage markers and chemokines and was primarily expressed in fibroblasts while only low levels were detected in adipocytes and macrophages. Administration of C1QTNF3 neutralizing antibodies inhibited macrophage accumulation in tumor-associated inguinal adipose tissue while tumor growth was unaffected. In line with this finding, C1QTNF3 exerted chemotactic actions on both M1- and M2-polarized macrophages *in vitro*. Moreover, C1QTNF3 treatment of M2-type macrophages stimulated the ERK and Akt pathway associated with increased M1-like polarization as judged by increased expression of M1-macrophage markers, increased production of nitric oxide, reduced oxygen consumption and increased glycolysis. Based on these results, we propose that macrophages are recruited to adipose tissue sites with increased C1QTNF3 production. However, the impact of the immunomodulatory effects of C1QTNF3 in adipose tissue remodeling warrants future investigations.

## 1 Introduction

Adipose tissue can dynamically alter its size, cellular composition, and metabolic function in response to hormonal and nutritional changes. This plasticity requires effective tissue remodeling processes in which macrophages are thought to play a key role. For instance, anti-inflammatory M2-type adipose tissue macrophages increase in response to fasting, weight loss or cold temperature, and are suggested to buffer excess lipids and stimulate thermogenesis (1–3). Interestingly, a recent study shows that resident macrophages protect against pathological adipose tissue remodeling in obesity (4). In contrast, increased accumulation of pro-inflammatory M1-type macrophages in obese adipose tissue is considered to contribute to chronic inflammation that causes insulin resistance (5, 6). Macrophages are also well known to infiltrate tumors. A high density of tumor-associated macrophages is correlated with worse prognosis for most cancers and unlike obese adipose tissue most macrophages in late-stage tumors are of the M2-type, which have been shown to stimulate tumor progression and metastasis (7–9). Clearly, macrophage subtypes exist through a continuum and the dichotomous M1-M2 model is often too simplistic to explain all the functional aspects (10–12).

Paracrine crosstalk between adipose tissue and cancer cells has been shown to enhance tumor growth (13–18), and the mechanical pressure from a growing tumor will also trigger substantial tissue remodeling in adjacent adipose tissue. To identify new adipose tissue-derived factors potentially important in subcutaneous adipose tissue remodeling and/or tumor progression, we compared the global gene expression profiles of breast cancer-associated and control inguinal/mammary white adipose tissue (IWAT) from lean and high fat diet (HFD)-induced obese female *C57Bl/6* mice. We chose to focus on upregulated secreted factors in breast cancer-associated IWAT that, to our knowledge, have not previously been studied in the context of adipose tissue remodeling and/or breast cancer progression. This approach led us to the cytokine C1Q and TNF related 3 (C1QTNF3, also called CTRP3, CORS26, cartducin and cartonectin) that belongs to the C1q/TNF-related protein-family. C1QTNF3 shares sequence homology with the insulin-sensitizing and anti-inflammatory adipokine adiponectin and is highly expressed in adipose tissue (19, 20). Since its discovery in 2001 (20), a number of experimental *in vitro* studies have been conducted and C1QTNF3 has been suggested to e.g. stimulate proliferation (21), inhibit LPS-induced inflammatory responses in fibroblasts, adipocytes and macrophages (22–25) and increase the secretion of adiponectin from adipocytes (21). Moreover, C1QTNF3 has been shown to exert beneficial effects on metabolism and inflammation *in vivo*; administration or transgenic overexpression of C1QTNF3 attenuated diet-induced hepatic steatosis and lowered glucose levels in Ob/Ob mice (26, 27) and C1QTNF3 knockout mice are more susceptible to collagen-induced arthritis in mice (28). Patients with type-2 diabetes have lower circulating C1QTNF3 levels (29) and visceral adipose tissue *C1qtnf3* levels are reduced in obesity/insulin resistant conditions (30–32), but less is known about *C1qtnf3* regulation in subcutaneous adipose tissue. Here, we found that IWAT *C1qtnf3* expression increases in response to both a growing a tumor and to HFD-induced obesity, and this *C1qtnf3* expression correlated with the expression of several macrophage markers and chemokines. We thus hypothesized that C1QTNF3 plays a role in macrophage regulation in breast cancer and/or subcutaneous adipose tissue remodeling. In line with this hypothesis, our data show that C1QTNF3 contributes to breast cancer-induced macrophage infiltration in IWAT. We found however no effect of C1QTNF3 neutralization on the macrophages within the tumor. Thus, IWAT C1QTNF3 affects macrophages locally in IWAT, but not in the adjacent tumor. *In vitro*, we demonstrate that C1QTNF3 is chemotactic for M1- and M2-macrophages and pushes M2 macrophages towards an M1-like phenotype. Thus, we propose that C1QTNF3 exerts immunomodulatory functions in a cell- and physiological state dependent manner.

## 2 Materials and Methods

### 2.1 Animals, Breast Cancer Model and IWAT Dissection

Female and male *C57Bl/6* mice, obtained from Charles River Laboratories (MA, U.S.A), were allowed to acclimatize for one week upon arrival. The mice were maintained on standard housing conditions of 12-hour light/dark cycle and temperature with *ad libitum* access to water and regular chow or high fat diet (HFD, 60% kcal from fat, D12492 from Research Diets, NJ, U.S.A.) as indicated.

At the age of about 20 weeks, IWAT of female mice were transplanted orthotopically with the breast cancer cell line E0771 (1×10^5^ cells in 50 µL) derived from a *C57Bl/6* mouse (33). Cells were suspended in an equal volume Matrigel (Matrigel Basement Membrane Matrix, Corning Inc, NY, U.S.A) and phosphate buffered saline (PBS). Sham control IWAT received Matrigel alone. The mice were euthanized 2-3 weeks after tumor transplantation, and sham control- and tumor-associated IWAT, and tumors were harvested. In brief, the inguinal lymph node was carefully removed. Thereafter, the tumor was excised containing a 1-2mm-sheet of surrounding adipose tissue. Tumor-associated IWAT were dissected out, and the adipose tissue sheet covering the tumor was carefully removed. Thus, some border material was discarded to avoid contamination. To ensure enough cells in the flow cytometry analysis, most IWAT was included while the most peripheral IWAT was removed in the RNA-sequencing experiment (**Supplementary Figure 1**). All experiments were approved by the regional Animal Ethics Committee in Gothenburg, Sweden.

### 2.2 Cell Lines and Cell Culture

The pre-adipocyte cell line 3T3-L1 (ZenBio, Durham, NC, U.S.A.) was maintained in supplemented DMEM growth media containing high glucose (4500 mg L^–1^), fetal bovine serum (10%, FBS Gold) and penicillin–streptomycin (1%, Thermo Fisher Scientific, MA, U.S.A) (34). The differentiation into adipocytes was carried out according to standard procedures (35). In brief, upon reaching sufficient confluency, cells were treated with a differentiation cocktail (1 μm dexamethasone, 850 nM insulin, and 0.5 mM 3-isobutyl-1-methylxanthine) for 2 days. Thereafter the media was interchanged with fresh media containing a second differentiation cocktail (850 nM insulin) for additional 2 days upon the media was returned to regular supplemented DMEM. The 3T3-L1 adipocytes maturity was determined by the prevalence of lipid droplets and then the cells were assayed, usually between 8 to 10 days from start of differentiation.

The E0771 cell line was maintained in supplemented RPMI containing glucose (2000 mg L^–1^), supplemented with fetal bovine serum (10%, FBS Gold), penicillin–streptomycin (1%), HEPES (10 mM) and sodium pyruvate (1 mM, Thermo Fisher Scientific, MA, U.S.A.).

### 2.3 L-929 Conditioned Media

The L-929 fibroblast cell line (ATCC, VA, U.S.A) was cultured in DMEM containing high glucose (4500 mg L^–1^) supplemented with 10% FBS, 1% Non-essential amino acids (NEAA, Thermo Fisher Scientific, MA, U.S.A.), 1% penicillin–streptomycin during 4 days upon the resulting media was harvested, sterile filtered (0.2 µM) and stored at -80°C until use.

### 2.4 Isolation of Bone Marrow Derived Stem Cells, Macrophage Differentiation and Activation

Femur and tibia bones were dissected and rinsed in ethanol. The ends of the bones were then cut off using a bone scissor. The bone marrow was flushed out with RPMI using a syringe and needle (26G). Cells of the bone marrow were further dissociated using a wider gauged needle (18G) and then pelleted (1000 rpm, 4 minutes at room temperature). Red blood cells were then depleted by adding lysis buffer (155 mM NH_4_Cl, 10 mM KHCO_3_, 0.1 mM EDTA, pH 7.2, for 5 minutes at room temperature), thereafter the reaction was impeded by adding cell culture media, and bone marrow derived cells pelleted (1000 rpm, 4 minutes at room temperature). The cells were resuspended in DMEM growth media containing low glucose (1000 mg L^–1^) supplemented with 30% L-929 conditioned media, 1% NEAA and allowed to differentiate for 6 days. At day 6, the cells were re-seeded into appropriate experimental cell culture plates and the differentiation continued for another two days upon the macrophages were activated: M1-type (5 ng/mL LPS and 12 ng/mL interferon (IFN) -γ, Thermo Fisher Scientific, MA, U.S.A.) and M2-type [10 ng/mL interleukin (IL)-4, Thermo Fisher Scientific] polarization was induced during respectively, 24 and 48 h.

### 2.5 Adipose Tissue *Ex Vivo* Culture to Measure C1QTNF3 Secretion

Whole adipose tissue was collected in PBS (5 mM glucose) at room temperature. Tissues were manually severed with scissors in petri dishes (5-10 mg fragments). Thereafter, samples were filtered (100 µm mesh) and washed with PBS to remove cellular debris and blood. Samples were then incubated in 12-well plate pre-filled with RPMI (1 mL, serum-free, 2.5 nM dexamethasone, HEPES 10 mM, 0.5 nM insulin, sodium pyruvate 1 mM) for 6 hours. Protease inhibitors were added during the incubation time to minimize protein degradation (Complete™, Mini, EDTA-free Protease Inhibitor Cocktail, Sigma-Aldrich, MO, U.S.A). The tissues were snap frozen in liquid nitrogen while the supernatants were transferred to Eppendorf tubes and centrifuged (1000 rpm, 4 minutes) to pellet cellular debris upon the pure samples were snap-frozen. All samples were stored at -80°C until analysis.

### 2.6 C1QTNF3 Recombinant Protein

Recombinant human embryonic kidney 293 cells-produced mouse complement C1q tumor necrosis factor-related protein (C1QTNF3) dissolved in sterile Tris-HCL (10 mM), EDTA (1 mM), glycerol (10%) at pH 8 (MyBioSource, CA, U.S.A.) was used in all C1QTNF3 treatment experiments (controls received only this buffer).

### 2.7 C1QTNF3 Antibody Treatment *In Vivo*

Obese mice were given goat anti-mCTRP3/C1QTNF3/CORS26 (AF2436, Research and Diagnostic Systems, Inc. MN, U.S.A.) intraperitoneally (0.5 mg/kg) by injection every second day for 14 days after tumor transplantation. An antibody of the same isotype (polyclonal goat IgG) served as control.

### 2.8 C1QTNF3 Measurement in Supernatant and Serum

C1QTNF3 levels in supernatant from adipose tissue culture and in serum were determined by a commercial ELISA according to the manufacturer’s protocol (CUSABIO, Houston, TX, U.S.A.).

### 2.9 Mitochondrial Respiration

#### 2.9.1 E0771 Cells, and 3T3-L1 Pre- and Adipocytes

Seahorse cell culture microplates (Agilent Technologies, CA, U.S.A.) were seeded with either E0771 cells (at 40 000/well) or 3T3-L1 cells (at 10 000/well, and adipogenesis was induced as described above). Upon reaching maturity, the cells were treated with C1QTNF3 (1µg/ml) for 24 h, upon the mitochondrial function was assayed with a Cell Mito Stress Test in a Seahorse XFe96 Analyser (Agilent Technologies). In brief, the test utilizes inhibitors of the mitochondrial oxidative phosphorylation: ATPase inhibitor (1 μM oligomycin), proton uncoupler (0.5 μM FCCP) and complex I and III inhibitor (0.5 μM rotenone and antimycin A), are added in the respective order. The obtained oxygen consumption rate (OCR) values in response to these different inhibitors were used to calculate basal, maximal, non-mitochondrial, ATP production- and proton leak-linked respiration, as well as spare respiratory capacity. All Seahorse experiments have been repeated three times and the average in each experiment is based on 5-10 replicates per group.

#### 2.9.2 Bone Marrow Derived Macrophages

Seahorse cell culture microplates were seeded with bone marrow derived macrophages (BMDM) at day 6 of maturation (at 80 000/well). The Cell Mito Stress Test was conducted similarly as described for above for 3T3-L1 cells except for compound concentrations (1 µM oligomycin, 2 µM FCCP, 0.5 µM rotenone and 0.5 µM antimycin A).

### 2.10 Glycolytic Function

The glycolytic function, as judged by extracellular acidification rate (ECAR), was measured in cultured BMDM by the Glycolytic Stress Test (Agilent Technologies) in a Seahorse XFe96 Analyser (Agilent Technologies, CA, U.S.A.). In brief, this test utilizes the addition of glucose (10 mM), oligomycin (1 µM) and a competitive inhibitor of glucose (50 mM 2-Deoxy-D-glucose), in the respective order. The obtained ECAR values were used to calculate glycolysis, glycolytic capacity, glycolytic reserve, and non-glycolytic acidification.

### 2.11 RNA-Isolation, cDNA-Synthesis, and Quantitative Real-Time PCR

RNA from tissue and cell lysates were isolated and purified using ReliaPrep RNA Cell MiniPrep System (Promega Corporation, WI, U.S.A) according to manufacturer’s protocol, except for processing adipose tissue, which requires removal of lipids prior the chloroform extraction. The concentration of the total RNA was determined by a NanoDrop (Thermo Fisher Scientific, MA, U.S.A.), and cDNA was generated through reverse transcription (500-1000 ng RNA) using a priming mixed strategy, according to manufacturer’s protocol (qScript Flex cDNA Synthesis Kit, Quanta Biosciences, MA, U.S.A). The gene expression was quantified through quantitative real-time PCR using SYBR-green (Fast SYBR^®^ Green Master Mix, Applied Biosystems, CA, U.S.A) and the relative ΔΔC_q_ method with either *Actb* or *Tbp* as endogenous control. The stability of the reference genes was determined from an array of housekeeping genes using NormFinder. Primers (**Supplementary Table 1**) were used at a concentration of 0.5 μM.

### 2.12 RNA-Sequencing and Analysis

RNA was isolated from adipose tissue (located as described in **Supplementary Figure 1** **)** and provided to the Bioinformatics Core Facility at the Sahlgrenska Academy, University of Gothenburg. The samples were enriched for RNA transcripts by depleting the ribosomal RNA (RiboMinus Technology, Thermo Fisher Scientific). The concentrated samples were then submitted to cDNA synthesis upon the adenylated 3’ ends were allowed to ligate with adapters, amplified and clustered on a chip (bridged PCR amplification). The clusters were then extended with single fluorescent labeled nucleotides and sequenced by imaging (reversible terminator sequencing, Illumina, CA, U.S.A.).

Quality assessment of the sequence reads was performed by generating QC statistics with FastQC (http://www.bioinformatics.bbsrc.ac.uk/projects/fastqc). RNA-sequencing reads were filtered and trimmed with prinseq (0.20.3). Mapping towards the mouse genome (mm10 from UCSC) was performed with the STAR aligner (2.4.0f) with default parameters (36). Raw gene counts were generated with HTSeq (https://htseq.readthedocs.io/en/release_0.10.0/). DESeq2 was then used to find differentially expressed genes (37). Unsupervised clustering of gene expression profiles was calculated using k-means on genes that were expressed significantly different between chow-fed control adipose tissue and chow-fed tumor associated adipose tissue. RNA-sequencing data have been deposited in NCBI’s Gene Expression Omnibus (38) and are accessible through GEO Series accession number GSE201316.

### 2.13 Primary Adipocyte and Stroma-Vascular Cell Isolation

Primary adipocytes and stroma-vascular cells (SVF) were isolated as in (39). Briefly, after collection, adipose tissue was minced and digested with 1 mg/ml collagenase type 2 (Sigma Aldrich, MO, U.S.A) in a buffer containing 123 mM NaCl, 5 mM KCl, 5 mM CaCl2 1.2 mM KH2PO4, 1.2 mM MgSO4, 25 mM HEPES, 2 mM glucose, 200 nM adenosine, 1.5% BSA. After digestion (30 min at 37°C), cells were filtered through 100 µm mesh and centrifuged (5min, 500g). Primary adipocytes were collected (floating fraction) and SVF (pelleted fraction) was further processed for red blood cell removal. Red blood cells were removed with RBC lysis buffer (Biolegend, CA, USA) treatment for 2 min at RT. After final wash, cells were resuspended in DMEM 10% FBS, counted on automatic cell counter (Countess 2, Thermo Fisher Scientific, MA, U.S.A.) and stained for flow cytometry analysis.

### 2.14 Magnetic Activated Cell Sorting

After IWAT and GWAT SVF isolation, cells were further separated into different subsets with magnetic activated cell sorting on OctoMACS™ separator (Miltenyi Biotec, Germany) per manufacturer’s instructions. Briefly, cells were incubated with anti-F4/80 MicroBeads UltraPure (Miltenyi Biotec, Germany) for 15 min in dark and on ice. After two washes in MS buffer (PBS, 0.5% BSA, 2mM EDTA), cells were loaded onto pre-rinsed MS columns (Miltenyi Biotec, Germany) placed in the magnetic field. Columns were rinsed two more times with MS buffer, and entire flow-through containing unlabeled cells was collected. Then, column was removed from magnetic separator and placed on collection tube. After adding MS buffer, magnetically labelled cells were flushed out with plunger. For fibroblast separation, F4/80^-^ cells were first depleted from leukocytes with anti-CD45 MicroBeads (Miltenyi Biotec, Germany). Negative fraction was subsequently incubated with anti-CD90.2 MicroBeads (Miltenyi Biotec, Germany). Cells were resuspended in Qiazol (Qiagen, Germany) for RNA isolation. Purity of all sorted cells (macrophages, F4/80^+^), fibroblasts (F4/80^-^CD45^-^CD90.2^+^) and all negative cells was confirmed with quantitative real-time PCR.

### 2.15 Flow Cytometry

Adipose tissue SVF cells were isolated as described above and cell suspensions from spleen and E0771 tumors were prepared by pushing tissue pieces though nylon mesh using a syringe plunger. Cells were resuspended in Fluorescence-Activated Cell Sorting (FACS) buffer and transferred to a round bottom 96-well plate. Samples were blocked with FC-block (4°C, 30 min, darkness) prior to staining with antibody panel (**Supplementary Table 2**). Samples were pelleted (1000 rpm, 3 min) and resuspended in FACS buffer to remove excess antibodies. Thereafter, samples were fixated and permeabilized prior to staining with intracellular antibodies and then resuspended in FACS buffer to remove additional excess antibodies before analysis (FACSAria and FacsCanto II; BD Biosciences, Franklin Lakes, NJ, USA). Adipose tissue macrophages (F4/80^+^CD11b^+^) were subgrouped by their CD206 and NOS2 expression (**Supplementary Figure 2**). For *in vitro* studies, C1QTNF3 (1 µg/ml) was added to BMDM during polarization stimulus. Supernatants were collected for nitric oxide measurements and kept on -80°C until analysis. Cells were collected and stained with LIVE/DEAD™ Fixable Blue Dead Cell Stain Kit (Thermo Fisher Scientific, MA, U.S.A.) followed by staining with surface and intracellular antibodies as above. For proliferation studies, differentiated BMDM (M0, M1 and M2) were treated with C1QTNF3 (1 µg/ml) for 24h. EdU (5-ethynyl-2’-deoxyuridine, A1004, Invitrogen) in concentration of 10 µM was added for the last 3h of culture. Cells were collected, stained for surface antibodies, EdU with EdU-click it assay (Thermo Fisher Scientific, MA, U.S.A.), intracellular antibodies and analyzed with flow cytometry. Gates were set with Fluorescent Minus One controls. Flow cytometry data was analyzed using FlowJo software version 10.6.0 (FlowJo, LLC, OR, U.S.A.).

### 2.16 Migration Assay

M0, M1 and M2-polarized BMDMs were seeded (15000/well) on transwell 96-well permeable supports with 8 µm pore size (Corning) in serum-free media (DMEM 0.5% BSA). C1QTNF3 (1µg/ml), MCP1 (positive control, 100 ng/ml) or serum-free media (negative control) were added in the bottom part of the 96-well plate. After overnight incubation, inserts were washed twice with PBS, and the cells on the inside of the insert were gently removed with moistened cotton swabs. Cells on the lower surface of the membrane were stained with crystal violet solution (Sigma Aldrich) for 15 min. Excess dye was removed with extensive washing with dH_2_O. After drying, bounded crystal violet was eluted with 33% acetic acid and the absorbance was measured at 590 nm on Spectramax i3x multiplate reader (Molecular Devices, CA, U.S.A.).

### 2.17 Western Blot

Cells were homogenized in lysis buffer (RIPA containing 150 mM sodium chloride, 1.0% Triton X-100, 0.5% sodium deoxycholate, 0.1% sodium dodecyl sulfate, 50 mM Tris, pH 8.0) and incubated on ice for 1 hour. Thereafter, the samples were centrifuged (12 000 rpm, 20 min, 4°C). The supernatants were collected, and the protein concentration was determined by the Pierce BCA Protein Assay (Thermo Fisher Scientific, MA, U.S.A.). The samples were then further processed by adding reducing agent (4x Laemmli Sample Buffer with β-mercaptoethanol, Bio-Rad Laboratories, CA, U.S.A.) and boiled to destruct the disulfide bonds (95°C, 5 min). Equal amounts of samples were separated on precast gels (Criterion TGX stain-free precast gel, Bio-Rad Laboratories, 100 V, 2 hours). The separated proteins were visualized using the stain free system and transferred to polyvinylidene difluoride (PVDF) membranes (Trans-Blot turbo Mini/Midi PVDF transfer pack, Bio-Rad Laboratories, CA, U.S.A.). To measure the activity of MEK/ERK1/2 and AKT signaling pathways the PVDF membrane was first blocked (TBS-Tween, BSA 5%, 30 min, room temperature). The primary antibodies (**Supplementary Table 3**) were added and allowed to incubate overnight at 4°C. Thereafter, a secondary antibody was added (room temperature, 60 min) upon the membranes were visualized using the ChemiDoc Imaging System (Bio-Rad Laboratories, CA, U.S.A.). The protein size was confirmed using a protein standard (Precision Plus Protein™ Dual Color Standards, Bio-Rad Laboratories, CA, U.S.A.). Background was subtracted prior data analysis, which was performed in Image Lab 6.0.1 (Bio-Rad Laboratories, CA, U.S.A.).

### 2.18 Nitric Oxide Measurements

Supernatants were transferred into Costar 96-well black plate with clear bottom (MERCK, Germany) in duplicates. 4,5-Diaminofluorescein (Sigma-Aldrich, MO, U.S.A.) was added in the final concentration of 10 µM. Fluorescence was measured on Spectramax i3x multiplate reader (Molecular Devices, CA, U.S.A.) with excitation wavelength of 495nm and emission wavelength of 525nm. Values were corrected with control sample (pure media).

### 2.19 Statistical Analysis

Student’s t-test was used for comparisons between two groups, and 1-way and 2-way ANOVA were used for comparisons between several groups and repeated measurements. Dunnett multiple comparison test for one-way ANOVA and Šidák correction for two-way ANOVA were used when multiple comparisons were done within the same analysis. The square of the Pearson’s correlation coefficient was used to measure the linear relation between variables. A p-value of <0.05 was considered significant and data are presented as mean ± SEM.

## 3 Results

### 3.1 Inguinal/Mammary Adipose Tissue C1QTNF3 Levels Increase in Response to Breast Cancer

**Figure 1:**
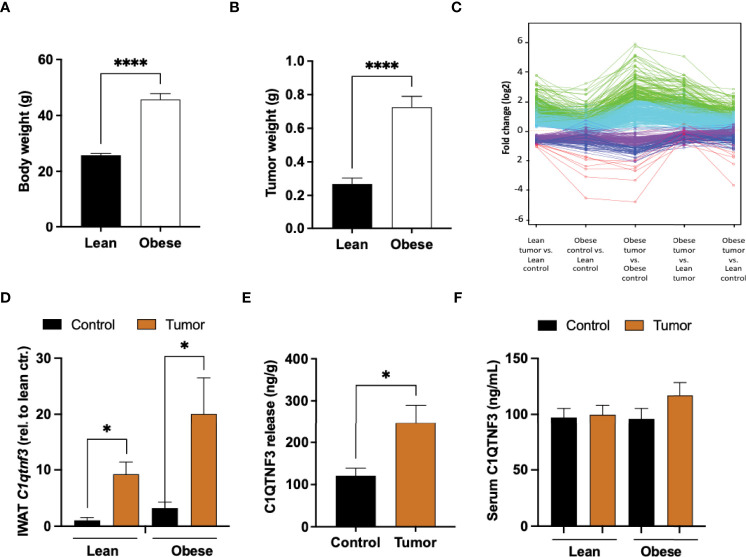
Increased *C1qtnf3* levels in tumor-associated adipose tissue. **(A)** Body weight and **(B)** E0771 tumor weight of lean and HFD-induced obese C57BL/6 female mice (N = 18-20/group). **(C)** Unsupervised cluster analysis of genes (detected by RNA-sequencing) that were significantly different between cancer-associated and control (sham) adipose tissue in chow-fed lean mice: Green cluster includes genes that were most upregulated in tumor-associated adipose tissue and where this regulation was enhanced by obesity (**Table 1**). **(D)** *C1qtnf3* mRNA levels in control (sham) and tumor-associated lean and obese inguinal white adipose tissue (IWAT) as judged by qPCR (N = 4-6/group), **(E)** C1QTNF3 protein release from control and tumor-associated obese female IWAT (N = 9/group), and **(F)** serum C1QTNF3 levels in lean and obese female mice with or with E0771 breast tumor (N = 5-10/group). *p < 0.05, ****p < 0.0001 for the indicated comparisons.

Female mice, fed either regular chow or high fat diet (HFD) for 16 weeks, were injected with E0771 cancer cells resuspended in PBS mixed with Matrigel into their left inguinal/mammary fat pad (tumor-associated IWAT) while their right inguinal fat pad (control IWAT) received PBS-Matrigel solution alone. Tumors, tumor-associated and control IWAT were collected two weeks after cancer cell injections (**Supplementary Figure 1**). As expected, the HFD-fed mice developed obesity and exhibited almost three times larger tumors than the chow-fed lean controls (**Figures 1A, B** **)**. This difference in E0771 tumor progression between lean and obese mice agrees with previous studies (18, 40). The global gene expression profiles of cancer-associated and control IWAT were compared using RNA-sequencing. To identify similarly regulated genes, we performed unsupervised cluster analysis of genes that were significantly different between cancer-associated and control IWAT in chow-fed lean mice. This analysis resulted in the identification of five clusters where genes within each cluster were co-regulated across the four groups (**Figure 1C** and **Supplementary Table 4**). Thereafter, we chose to take a closer look on the cluster where the genes were upregulated by tumor presence and where this regulation was enhanced by obesity/HFD (green-colored cluster in **Figure 1C**). Within this cluster, we found several genes that are known to be involved in tumor progression e.g., the acute phase reactant *Saa3*, several chemokines (*Ccl2, Ccl7, Ccl8, Ccl12*) as well as several macrophage markers such as *Emr1* (*F4/80*), *Ccr5*, *Itgam* (*Cd11b*) and *CD68*. One of the most regulated genes (as judged by fold change between control and tumor-associated adipose tissue) within this cluster was however *C1qtnf3* (**Table 1** and **Figure 1D**), an adipokine that to our knowledge has not previously been studied in the context of breast cancer progression. Importantly, the increased *C1qtnf3* expression in tumor-associated IWAT was associated with increased release of C1QTNF3 protein (**Figure 1E**). The circulating levels of the C1QTNF3 were however unaffected by both diet-induced obesity and experimental breast cancer (**Figure 1F** **).**

**Table 1:** Green cluster: Genes most upregulated in tumor-associated compared to control inguinal/mammary adipose tissue. Red numbers indicate significant log2-fold changes.

ADIPOSE TISSUE	LEAN	OBESE	CONTROL	TUMOR
GENE ID	Tumor vs. Control	Tumor vs. Control	Obese vs.Lean	Obese vs. Lean
*Slfn4*	3.75	5.02	0.67	1.94
*Ccl7*	3.72	3.09	1.93	1.30
*Has1*	3.31	3.76	1.34	1.79
*Ccl8*	3.17	2.79	0.08	-0.31
*C1qtnf3*	3.15	3.51	1.22	1.57
*Ly6c2*	3.12	2.22	1.65	0.75
*Chi3l3*	3.07	3.08	2.79	2.79
*Oas3*	3.05	3.23	0.53	0.70
*Ms4a4c*	2.81	1.93	2.43	1.55
*Ccl2*	2.80	3.14	1.98	2.32
*Saa3*	2.79	2.07	2.72	2.00
*Clec4d*	2.76	2.57	2.46	2.27
*Ccl12*	2.72	3.29	0.17	0.73
*Cilp*	2.71	1.90	1.64	0.83
*Clec4n*	2.71	1.89	1.17	0.36
*Oas1g*	2.57	2.80	0.19	0.43
*Slfn1*	2.53	1.99	0.99	0.46
*Fcgr1*	2.47	3.32	0.26	1.10
*Bst1*	2.36	1.98	1.72	1.33
*Nxpe5*	2.34	2.77	0.17	0.60

### 3.2 *C1qtnf3* Is Primarily Expressed in the Stromal Vascular Fraction of Adipose Tissue and Displays an Adipose Depot-Specific Response to HFD-Induced Obesity

**Figure 2:**
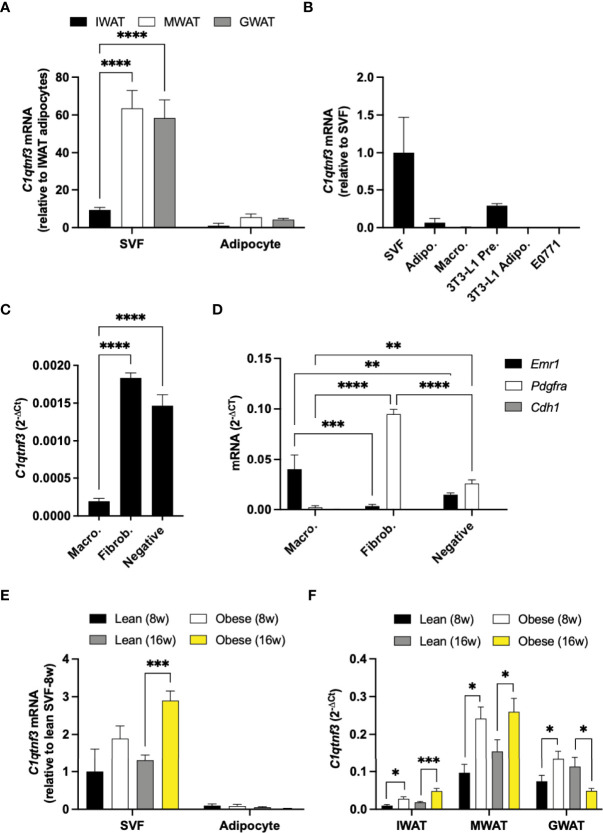
*C1qtnf3* is primarily expressed in the stromal vascular fraction of adipose tissue and displays an adipose depot-specific response to HFD-feeding. **(A)** *C1qtnf3* expression in the stromal vascular (SVF) and adipocyte fraction from inguinal (IWAT), mesenteric (MWAT) and gonadal white adipose tissue (GWAT) from unchallenged male mice on chow (N = 4/group, 2-way ANOVA: F_cell type_ (1, 18) = 77.11, p < 0.0001). **(B)** *C1qtnf3* expression in IWAT SVF, primary IWAT adipocytes, peritoneal macrophages, 3T3-L1 preadipocytes, 3T3-L1 adipocytes and E0771 breast cancer cells (N = 4-6/group). **(C)** *C1qtnf3* expression in IWAT macrophages (F4/80^+^), fibroblasts (F4/80^-^CD45^-^CD90.2^+^) and in remaining F4/80^-^CD45^-^CD90.2^-^ SVF cells, and **(D)** analysis of cell population purity by qPCR. **(E)** Female IWAT SVF and adipocytes in response to 8- and 16-week (w) high fat diet (HFD) feeding (N = 10/group, 2-way ANOVA: F_cell type_ (1, 54) = 51.99, p < 0.0001). **(F)** IWAT, MWAT and GWAT *C1qtnf3* expression in response to 8- and 16-week (w) HFD feeding in male mice (N = 6-10/group). *p < 0.05, **p <0.01, ***p < 0.001, and ****p < 0.0001 for the indicated comparisons.

To further characterize the regulation of *C1qtnf3*, we quantified *C1qtnf3* levels in different fat depots and cell types. In unchallenged male mice, *C1qtnf3* mRNA levels were higher in gonadal (GWAT) and mesenteric white adipose tissue (MWAT) than in IWAT, and *C1qtnf3* was much more abundant in the stromal vascular fraction (SVF) than in the adipocyte fraction (**Figure 2A**). In line with a previous study (41), cultured 3T3-L1 preadipocytes (fibroblasts) displayed relatively high expression of *C1qtnf3* and this expression was substantially reduced in mature 3T3-L1 adipocytes (**Figure 2B**). The *C1qtnf3* expression was even lower in peritoneal macrophages than in 3T3-L1 adipocytes and undetectable in E0771 breast cancer cells (**Figure 2B**). To further clarify the *C1qtnf3* expression pattern, we analyzed the expression in sorted adipose tissue resident macrophages (F4/80^+^cells) and fibroblasts (F4/80^-^CD45^-^CD90.2^+^ cells) from IWAT and GWAT. This confirmed relatively low expression in macrophages compared to fibroblasts, but there was also relatively high expression in cells negative for all markers (**Figures 2C, D** and **Supplementary Figures 3A, B**). Moreover, the IWAT SVF expression of *C1qtnf3* was increased in HFD-induced obese female and male mice (**Figure 2E** and **Supplementary Figure 3C**), while the adipocyte *C1qtnf3* expression remained low and unaltered by HFD/obesity. To further elucidate the regulation of adipose tissue *C1qtnf3*, we analyzed the *C1qtnf3* levels in IWAT, MWAT and GWAT after 8- and 16-week-chow versus HFD feeding in male mice. After 8 weeks of HFD-feeding, the *C1qtnf3* expression was increased in IWAT, GWAT and MWAT and the expression remained elevated in IWAT and MWAT also after 16 weeks of HFD-feeding. In contrast, 16-week HFD-feeding was associated with reduced GWAT *C1qtnf3* expression (**Figure 2F**). As expected from the cluster analysis (**Figure 1C**, **Table 1**), the expression of *C1qtnf3* and the pan-macrophage marker *Emr1* were positively correlated in IWAT (R^2 =^ 0.53, p<0.0001) and MWAT (R^2 =^ 0.58, p<0.0001). However, no such correlation was found in GWAT (R^2 =^ 0.02, p=0.54). There was no impact of 8- or 16-week-HFD-feeding on circulating C1QTNF3 levels (**Supplementary Figure 3D**).

### 3.3 Antibody-Mediated Blockage of C1QTNF3 Reduces the Infiltration of Macrophages Into Tumor-Associated Adipose Tissue

**Figure 3:**
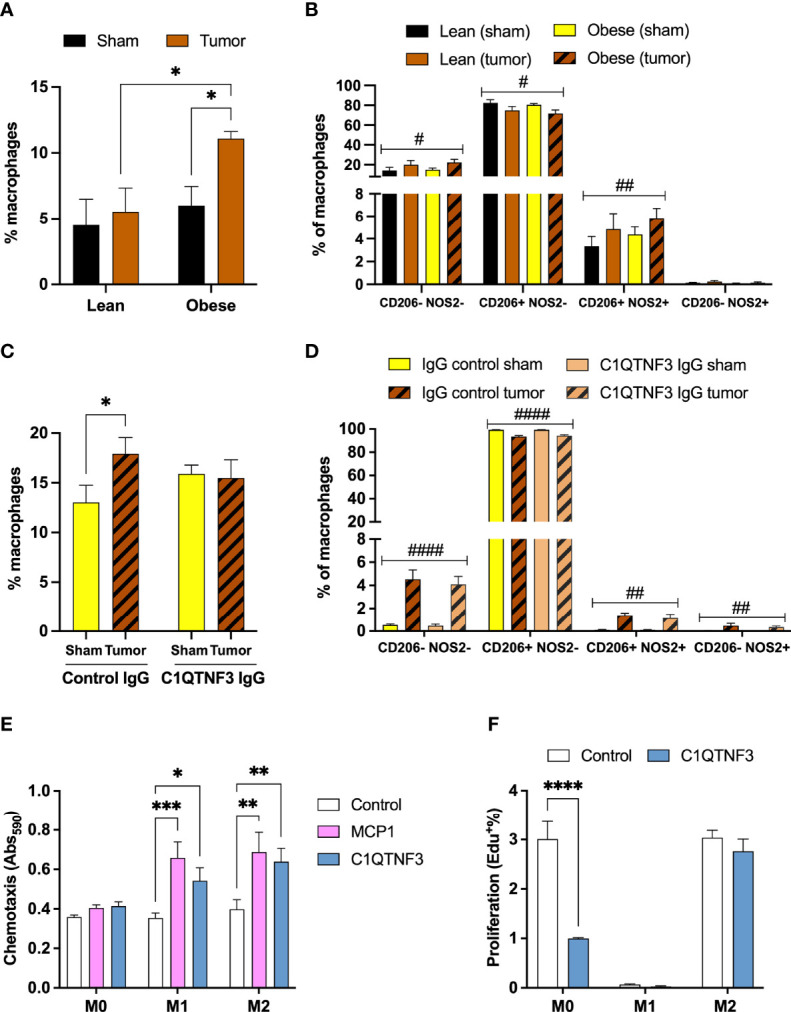
C1QTNF3 neutralization inhibits tumor-induced macrophage infiltration in adjacent inguinal adipose tissue in obese mice. **(A)** Total macrophages (% F4/80^+^CD11b^+^ of viable cells) and **(B)** % CD206^-^NOS2^-^, % CD206^+^NOS2^-^ (M2-type), % CD206^+^NOS2^+^ (mixed M1-M2-type), % CD206^-^NOS2^+^ (M1-type) of total macrophages in control and tumor-associated IWAT from lean and HFD-induced obese female mice (N = 4/group, each IWAT sample was pooled from 2-3 mice, ^#^p < 0.05 and ^##^p < 0.01 for the effect of tumor in 2-way ANOVA) **(C)** Total macrophages in control and tumor-associated IWAT from isotype control and C1QTNF3-IgG treated HFD-induced obese female mice (N = 5-6/group, 2-way ANOVA: F_treatment x tumor_ (1, 9) = 9.222, p < 0.05). **(D)** % CD206^-^NOS^-^, M2-type, mixed M1-M2-type, and % M1-type of total macrophages in control and tumor-associated IWAT (N = 5-6/group, ^#^p < 0.05, ^##^p < 0.01 and ^####^p < 0.0001 for the effect of tumor in 2-way ANOVA). **(E)** MCP1 (100 ng/ml) and C1QTNF3 (1 µg/ml)-induced chemotaxis (N = 5-10/group) and **(F)** proliferation, measured as % of EdU incorporation, with or without C1QTNF3 (1 µg/ml) of M0-, M1- and M2-polarized bone marrow derived macrophages (N = 3-4/group). *p < 0.05, **p <0.01, ***p < 0.001, and ****p < 0.0001 for the indicated comparisons.

To study the potential role of C1QTNF3 in tumor immunity and/or progression, we first characterized the macrophage infiltration in E0771 tumors, tumor-associated and control IWAT and spleen of untreated lean and HFD-induced obese female mice (body weight: 23.9±0.5 vs. 36.0±2.1 g, p<0.0001). The macrophage infiltration (% CD11b^+^F4/80^+^ of viable cells) was similar between tumors from lean and obese mice, but obesity was associated with an increased proportion of mixed M1-M2-type (% CD206^+^NOS2^+^ of CD11b^+^F4/80^+^ cells, **Supplementary Figures 4A, B**). However, neither M2-type-(CD206^+^NOS2^-^) nor M1-type-(CD206^+^NOS2^-^) tumor associated macrophages were altered by obesity (**Supplementary Figure 4B**). In line with the global gene expression data (**Supplementary Table 4** **)**, there was an increased accumulation of macrophages in tumor-associated IWAT compared to control IWAT in obese mice, but this increase was not seen in chow-fed lean mice (**Figure 3A**). It is possible that we fail to detect tumor-induced IWAT macrophage accumulation in the lean setting as the flow cytometry analysis includes also adipose tissue that is more distant to the tumor (**Supplementary Figure 1**). 2-way ANOVAs show that HFD/obesity had no effect on IWAT macrophage abundance or polarization (**Figures 3A, B**), while tumor presence reduced the proportion of M2-type macrophages [F_tumor_ (1, 6)=11.8, p=0.01] and increased the proportion of mixed M1-M2-type macrophages [F_tumor_ (1, 6)=16.5, p=0.007] (**Figure 3B**). In addition, HFD/obesity increased the percentage of total splenic macrophages, but tumor presence had no effect on neither the macrophage abundance nor the subset distribution (**Supplementary Figures 4C, D**). Led by these data, we chose to study the role of C1QTNF3 on macrophage accumulation and tumor growth in the HFD-induced obese setting. To this aim, we treated HFD-induced obese E0771 tumor-bearing female mice with either C1QTNF3 or isotype control antibody. The mice weighed similar prior to the implantation of tumor and antibody treatment (control IgG: 44.4±0.7 g; C1QTNF3 IgG: 44.7±0.8 g) and at termination (control IgG: 42.7±0.8 g; C1QTNF3 IgG: 44.7±0.9 g). We found that mice treated with isotype control antibody displayed the expected increase in total macrophages (as % CD11b^+^F4/80^+^ of viable cells) in tumor-associated IWAT compared to control IWAT, but this increase was absent in C1QTNF3 IgG treated mice (**Figure 3C**). According to 2-way ANOVAs, tumor presence reduced the proportion of M2-type macrophages [F_tumor_ (1, 18)=123.4, p<0.0001], increased the proportion of both mixed M1-M2-type [F_tumor_ (1, 18)=40.8, p<0.0001] and M1-type macrophages [F_tumor_ (1, 18)=16.0, p=0.0008] (**Figure 3D**). However, C1QTNF3 IgG treatment had no effect on IWAT macrophage polarization (**Figure 3D**), and we saw no difference between treatment groups in total, M1- or M2-type macrophages in tumor and spleen (data not shown). The overall higher macrophage infiltration in IWAT in this experiment compared to the HFD-fed group in **Figure 3A** may be due to the difference in weight gain between these experiments. Moreover, glucose levels changed similarly between groups after tumor implant and antibody treatment (Δglucose: -0.52±0.7 vs. -0.13±0.6 mM, p=0.7) supporting the notion that C1QTNF3, under this experimental condition, acts locally rather than acting as a classical hormone. Finally, there was no difference in the average tumor weight between groups (**Supplementary Figure 4E**) and we detected no lung metastasis in any of the animals.

### 3.4 C1QTNF3 Increases Macrophage Chemotaxis and Alters Macrophage Proliferation in a Subtype-Dependent Manner

To elucidate the mechanism for the inhibitory action of C1QTNF3 neutralization on tumor-induced IWAT macrophage accumulation, we analyzed the effect of C1QTNF3 treatment on chemotaxis and proliferation in bone marrow derived macrophages. We found that C1QTNF3 treatment stimulated chemotaxis of both M1- and M2-type macrophages and a similar trend was seen also for naïve (M0) macrophages (**Figure 3E**). The effect of C1QTNF3 on proliferation depended on polarization; C1QTNF3 treatment for 24h inhibited the proliferation in M0, whilst not affecting the proliferation of M1- and M2-type macrophages (**Figure 3F**).

### 3.5 C1QTNF3 Enhances M1-Like Polarization in Cultured Macrophages

**Figure 4:**
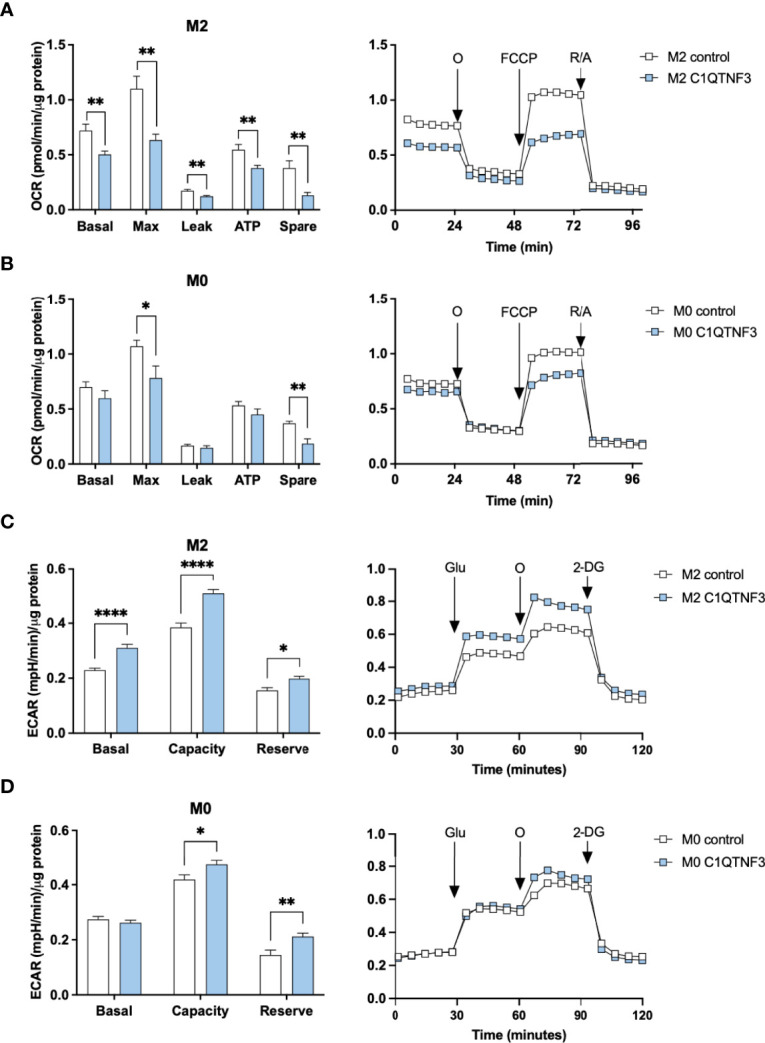
C1QTNF3 reduces respiration and increases glycolysis in bone marrow derived macrophages. Mitochondrial function, as determined by changes in basal respiration (Basal), maximal respiration (Max), proton leak-related respiration (Leak), ATP production-linked respiration (ATP) and spare respiratory capacity (Spare), was estimated from the oxygen consumption rate (OCR) of cultured bone marrow derived **(A)** M2- (N = 13-15/group) and **(B)** M0-type (N = 6-8/group) macrophages in response to subsequent addition of oligomycin (O), FCCP, and rotenone (R)/antimycin A as indicated in the right panels. Data are presented as OCR normalized to total protein levels. **(C)** Glycolytic function, as determined by basal glycolysis rate (Basal), glycolytic capacity (Capacity), and glycolytic reserve (Reserve), was estimated from the extracellular acidification rate (ECAR) in cultured bone marrow derived **(C)** M2- and **(D)** M0-type macrophages in response to subsequent addition of glucose (Glu), oligomycin (O) and 2-Deoxy-D-glucose (2-DG). Data are presented as ECAR normalized to total protein levels (N = 6-12/group). Prior to these OCR and ECAR measurements, the macrophages were treated with or without C1QTNF3 (1 µg/ml) for 48 h along with control (M0) or IL-4 to induce M2-polarization. *p < 0.05, **p < 0.01, ****p < 0.0001 for the indicated comparisons.

**Figure 5:**
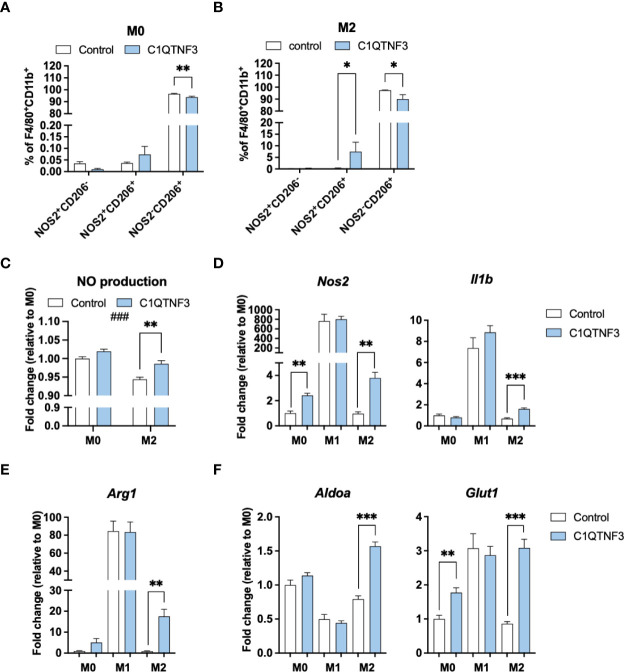
C1QTNF3 enhances M1-polarization and the expression of glycolysis markers in M0- and M2-type bone marrow derived macrophages. **(A, B)** Flow cytometry analysis of NOS2 and CD206 and **(C)** nitric oxide (NO) production in cultured bone marrow derived M0-, and M2-polarized macrophages (N = 3-5/group, ^###^p < 0.001 for the effect of treatment on NO production in 2-way ANOVA). The expression of the **(D)** M1-markers *Nos2* and *IL1b*, the **(E)** M2 marker *Arg1*, **(F)** the glycolysis-related genes *Aldoa*, and *Glut1* in cultured bone marrow derived M0-, M1- and M2-type macrophages (N = 6/group). NO production and gene expression data are presented as fold change relative to the M0 control. The macrophages were treated with or without or C1QTNF3 (1 µg/ml) for 48 h along with control (M0), IL-4 to induce M2-polarization, or LPS & IFNγ to induce M1- polarization. *p < 0.05, **p < 0.01, ***p < 0.001 for the indicated comparisons.

Based on the subtype-dependent effect of C1QTNF3 on macrophage proliferation, we hypothesized that C1QTNF3 also regulates other functional aspects in a subtype-dependent manner. To test this hypothesis, we treated bone marrow derived M0-, M1- and M2-type macrophages with mammalian-produced C1QTNF3 and analyzed oxygen consumption rate (OCR). Non-polarized macrophages were treated with C1QTNF3 during the entire 48h-period of their M1- and M2-type induction (M0-macrophages received either vehicle or C1QTNF3 alone). We found a rather dramatic effect of C1QTNF3 treatment on mitochondrial function in M2-type macrophages; C1QTNF3-treated M2-type macrophages displayed a reduction in most measured respiration parameters (**Figure 4A**). Similar results, albeit to a lesser extent, were seen in M0 and M1-type macrophages (**Figure 4B** and **Supplementary Figure 5A**). The smaller effect on respiration in M1-type macrophages is expected since these cells rely mostly on glycolysis. These data indicate that C1QTNF3 induces a metabolic shift from oxidative phosphorylation towards increased reliance on glycolysis for ATP-production in M2-type macrophages. Indeed, C1QTNF3-treated M2-type macrophages displayed increased basal glycolysis as judged by extracellular acidification rate (ECAR) (**Figure 4C**). Moreover, the glycolytic capacity and reserve were increased in both M0- and M2-type macrophages indicating that C1QTNF3 induces a macrophage phenotype that can meet an increased demand for ATP production (e.g. during an acute immune response) and/or can sustain in a competitive tumor microenvironment where nutrients are scarce (**Figures 4C, D**). This metabolic shift was echoed by a phenotype switch; C1QTNF3 treatment led to reduced % of CD206^+^NOS2^-^ cells in M0- and M2-polarized macrophages and an increased % of CD206^+^NOS2^+^ cells in M2-polarized macrophages (**Figures 5A, B**). Moreover, C1QTNF3-treated M2-type macrophages showed an increased production of nitric oxide (**Figure 5C**). An M1-like shift in polarization of M0 and M2-macrophages was also supported by an increased expression of M1-type polarization markers, such as Inducible nitric oxide synthase (*Nos2)* and Interleukin-1b (*Il1b*) (**Figure 5D**). Arginase-1 (*Arg1)*, an enzyme that typically is associated with M2-type macrophages, was also increased in C1QTNF3-treated M2-type macrophages (**Figure 5E**). While this may appear surprising, increased *Arg1* expression is seen in LPS-stimulated murine macrophages, possibly as a mean to attenuate NO-mediated inflammation (42, 43). Increased *Arg1* may thus in some contexts reflect increased pro-inflammatory signaling. In further support of the increased reliance of glycolysis in C1QTNF3-treated M2-type macrophages, the expression of several genes involved in glycolysis such as Aldolase A (*Aldoa*), Glucose transporter 1 (*Glut1*) and Hypoxia-inducible factor 1-alpha (*Hif1a*) was increased (**Figure 5F** and **Supplementary Figure 5B**).

**Figure 6:**
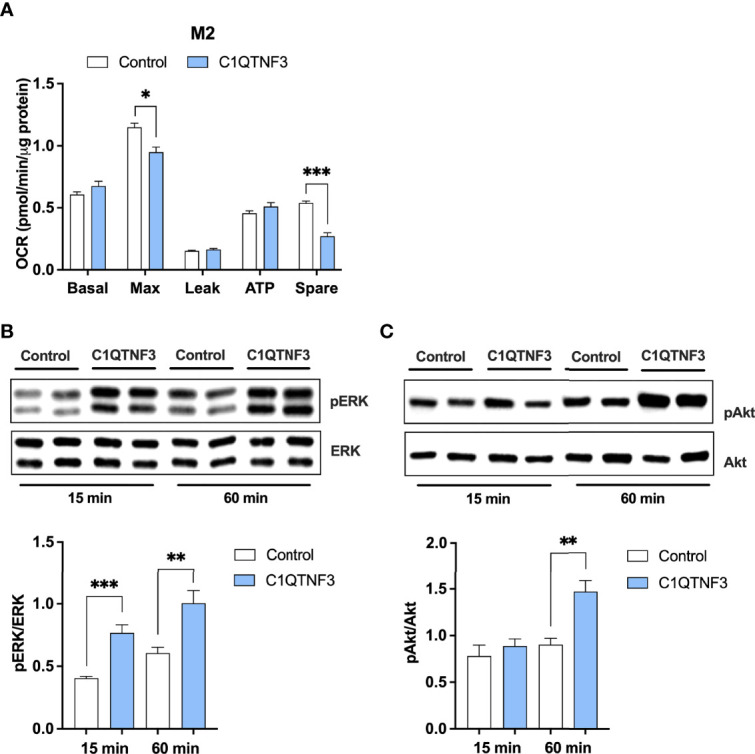
Short-term C1QTNF3 treatment activates the ERK- and Akt-pathways associated with reduced maximal respiration and reduced spare respiratory capacity in M2-type bone marrow derived macrophages. M2-type bone marrow-derived macrophages were treated with C1QTNF3 (1 µg/ml) for 1 h prior the **(A)** mitochondrial function analysis (N = 6-8/group). Representative blots and quantification of **(B)** phosphorylated (p)-ERK and total ERK and **(C)** p-Akt and total Akt in M2-type bone marrow-derived macrophages in response to 15- and 60-minute C1QTNF3 or vehicle treatment (N = 5/group). ERK and Akt activation are presented as a ratio of phosphorylated over total levels (N = 5/group). *p < 0.05, **p < 0.01, ***p < 0.001 for the indicated comparisons.

Given the observed rather potent effect of C1QTNF3 on macrophage metabolism, we asked whether C1QTNF3 exerts acute effect on respiration or whether the observed effects on respiration rely on long-term treatment during the activation process. To answer this question, we treated M0- and M2-type macrophages with C1QTNF3 for only 1h and assessed the effect on respiration. Although long-term treatment was more effective in this regard, we found that 1h C1QTNF3 treatment reduced the maximal respiration and spare respiratory capacity in M2-type macrophages (**Figure 6A** **).**

The negative effect of C1QTNF3 on macrophage respiration was to us unexpected findings and prompted us to also test the effect of C1QTNF3 on mitochondrial function in cultured pre-adipocytes, adipocytes, and E0771 breast cancer cells. In sharp contrast to the observed effect in macrophages, C1QTNF3 slightly increased the ATP production-coupled respiration in pre-adipocytes while there was no effect of C1QTNF3 on respiration either in adipocytes or in E0771 breast cancer cells (**Supplementary Figures 5C-E**). These data suggest that C1QTNF3 treatment affects the mitochondrial function in a cell-specific manner.

Previous research shows that C1QTNF3 can activate the MEK/ERK1/2 and PI3K/Akt pathways (26, 44), which can affect a wide a range of processes in macrophages including their viability, development, immunological and metabolic function. We therefore tested whether these pathways are regulated by C1QTNF3 also in our experimental settings. Indeed, we found that 15 minutes C1QTNF3 treatment increased the ERK-activation of cultured M2-type macrophages about 2-fold more than vehicle control treatment (**Figure 6B**), while there was no effect of C1QTNF3 on Akt-activation at that time point (**Figure 6C**). However, 60-minute C1QTNF3 treatment led to increased Akt-activation (**Figure 6C**).

## 4 Discussion

Here, we show that mRNA and protein levels of C1QTNF3 are upregulated in IWAT in response to breast cancer and that C1QTNF3-neutralizing antibody treatment inhibits the breast cancer-induced macrophage recruitment to IWAT in HFD-induced obese mice. Based on recombinant C1QTNF3 treatment experiments *in vitro*, we demonstrate that C1QTNF3 stimulates macrophage chemotaxis, and has profound inhibitory effects on M2-type macrophage respiration associated with increased glycolysis and increased M1-like polarization.

### 4.1 Orthotopic E0771 Breast Cancer – A Mouse Model That Can Reveal New Factors Involved in Subcutaneous Adipose Tissue Remodeling

We found that many macrophage-related genes that were up-regulated in tumor-associated compared to control adipose tissue in lean mice showed an even stronger tumor-induced upregulation in obese adipose tissue indicating that they may be involved in the enhanced tumor progression in obesity. However, contrary to this idea, we did not detect major changes in the percentage or polarization of tumor-associated macrophages between lean and HFD-induced obese mice. Moreover, our recent work shows that interactions between tumor and adjacent adipose tissue stimulate tumor growth to a similar degree in lean and obese mice (18), suggesting that the increased E0771 tumor growth in the obese mice primarily is due to systemic metabolic and/or endocrine alteration. Thus, we interpret the enhanced macrophage accumulation along with many of the enhanced transcriptional changes in obese tumor-associated IWAT rather as a consequence from the faster growing tumors than the cause. While this was disappointing from a breast cancer perspective, the orthotopic E0771 breast cancer model led us to C1QTNF3, thus showing its potential for identifying factors that are involved in subcutaneous adipose tissue remodeling.

### 4.2 Source of Adipose Tissue C1QTNF3

C1QTNF3 is considered an adipokine as it is highly expressed in adipose tissue. In line with some previous studies (19, 41), we found however, relatively low *C1qtnf3* expression in mature (primary and cultured) adipocytes, while the SVF of adipose tissue and cultured pre-adipocytes displayed higher levels. Moreover, our data suggest that fibroblasts, but not macrophages, constitute a major source for adipose tissue C1QTNF3. In support of this, publicly available single cell transcriptomics data show that the highest *C1qtnf3* expression within adipose tissue SVF is found in the mesenchymal stem cell cluster while other cell types such as lymphocytes and myeloid and endothelial cells express very modest levels (45). Thus, the *C1qtnf3* expression that we detect in CD45^-^F4/80^-^CD90.2^-^ cells may originate from mesenchymal cells expressing no or low levels of CD90.2.

### 4.3 Regulation of Adipose Tissue C1QTNF3 Levels

We found that the elevated *C1qtnf3* mRNA levels in tumor-associated IWAT was associated with a ~70% increase in released C1QTNF3 protein. However, serum levels of C1QTNF3 in tumor-bearing mice did not change, indicating that changes in local C1QTNF3 levels are more important than systemic in this context. Also, chronic HFD-feeding/obesity upregulated the IWAT *C1qtnf3* expression in both males and females, but to a lesser extent than in tumor-bearing mice. There was also no effect of chronic HFD-feeding on IWAT macrophage accumulation in females as judged by flow cytometry. In an additional HFD/obesity study in males, we found that *C1qtnf3* is regulated in an adipose depot-specific manner. 8- and 16-week HFD feeding were associated with upregulated *C1qtnf3* expression in both IWAT and MWAT. In contrast, 16-week HFD feeding led to reduced *C1qtnf3* levels in GWAT. To our knowledge, previous studies that have reported a downregulation of *C1qtnf3* in obese rodent adipose tissue have analyzed GWAT, and our data are thus in line with those observations (30–32). Long-term HFD feeding in male *C57Bl/6* mice is typically associated with increased adipocyte death and a dramatic increase in macrophage infiltration in GWAT (46) that is not seen in other fat depots. It is thus possible that the reduction in *C1qtnf3* in GWAT (but not in IWAT and MWAT) reflects this pathological tissue remodeling. A potent acute pro-inflammatory response within adipose tissue is essential for healthy adipose tissue expansion (47). Hence, the HFD-induced increase in IWAT *C1qtnf3* may play a role in this physiological acute pro-inflammatory response that must occur to effectively remodel the extracellular matrix and vasculature to accommodate the growing adipocytes in this depot. Overall, the immunological responses to HFD/obesity have been studied in detail in the GWAT in male mice. Much less is known about the immunological responses in other fat depots (and in females) – our data suggest that immune responses in subcutaneous adipose tissue may be much more dynamic than previously anticipated, and this warrants further investigations.

### 4.4 Metabolic and Immunological Effects of C1QTNF3

Pharmacological treatment with C1QTNF3, associated with a 3-fold elevation of circulating C1QTNF3 levels, lowers glucose levels in normal and insulin-resistant *ob/ob* mice (26). In this study, we were however unable to detect a significant effect C1QTNF3-neutralizing antibody treatment on glucose levels in HFD-fed obese breast cancer-bearing mice. We believe that this lack of glucose-regulatory effect of C1QTNF3 neutralizing antibodies is not surprising; firstly, breast cancer bearing mice did not display altered serum C1QTNF3 levels (this study) and mice lacking C1QTNF3 have unaltered glucose metabolism (48). Moreover, we found no effect on respiration in either cultured E0771 cancer cells or 3T3-L1 adipocytes although there was a small positive effect on respiration in cultured 3T3-L1 preadipocytes in line with a previous study (49). In contrast, we noticed a rather potent negative effect of C1QTNF3 on respiration in cultured bone marrow derived macrophages associated with increased M1-like polarization. The effector function of macrophages is linked to their metabolic function, where M1-type polarization parades with increased aerobic glycolysis, while M2-type polarization is associated with increased oxidative phosphorylation (10, 16). Our data is thus well in line with this concept; C1QTNF3 reduces the capacity for respiration in macrophages and thereby suppresses full polarization towards the oxidative M2-type phenotype (50, 51). The different response in different cell types may depend on the physiological context, whether the cells express a C1QTNF3 receptor or not, and what signaling pathways that dominates in respective cell type.

Previous research suggests that C1QTNF3 exerts anti-inflammatory effects (22, 23, 25, 28), and we were therefore initially surprised to find that antibody-mediated blockage of C1QTNF3 reduced the tumor-induced macrophage accumulation along with the chemotactic and pro-inflammatory effects of C1QTNF3 on bone marrow derived macrophages *in vitro*. On the other hand, our study shows that C1QTNF3 expression is regulated in a similar manner as many pro-inflammatory chemotactic factors in breast cancer-associated IWAT. Our results are also in line with a recent clinical study where adipose tissue *C1qtnf3* levels correlated positively with levels of *Tnfa*, *Ilb* and *Ccl2* (52). Moreover, neither genetic overexpression nor knockout of C1QTNF3 alters the GWAT and the systemic inflammatory response to a sublethal dose of LPS *in vivo*, and mice overexpressing C1QTNF3 display *increased* levels of several chemokines, although this difference between genotype disappears when animals are challenged with HFD/obesity (53). In the obese state, C1QTNF3 overexpression is instead associated with reduced serum levels of IL‐5 and TNF‐α, while soluble gp130 levels increased (53). Thus, C1QTNF3 may both enhance and reduce pro-inflammatory responses dependent on the immunological setting. Nevertheless, our results may be particularly difficult to reconcile with a study by Lin and colleagues where wheat germ-produced C1QTNF3 dose-dependently inhibited TNF-α, IL-6, MCP1, MMP9 and IL-1β release in LPS-stimulated THP-1 macrophages and mouse peritoneal macrophages (24). In our hands, we saw only small effects of C1QTNF3 treatment on LPS/IFNγ-stimulated bone marrow derived macrophages, while a M1-like phenotype was induced in IL-4-stimulated bone marrow derived macrophages. One possible reason underlying this discrepancy may be that bone marrow derived macrophages display a different phenotype in the context of C1QTNF3 treatment than other macrophage *in vitro*/*ex vivo* models (54). Also, we did not detect an effect of C1QTNF3 neutralization on macrophage polarization *in vivo*. It is possible that we fail to detect such an effect as C1QTNF3 attracts both M1- and M2-type macrophages. Alternatively, the effect of C1QTNF3 on macrophage polarization is outruled by more potent macrophage polarizing factors in tumor-associated HFD-induced obese IWAT. Besides differences in macrophage types and experimental settings, the dose, and the source of the recombinant C1QTNF3 protein may play a role. In this study, we have used a relatively high dose (1 µg/ml) of mammalian cell-produced mouse C1QTNF3 resulting in mammalian-type posttranslational modifications such as glycosylation that allows for correct folding and subsequent multimerization into higher-order oligomers (presumably trimeric, hexameric and high molecular weight oligomers) (19). Although the significance of posttranslational modifications and the different C1QTNF3 oligomeric forms is yet to be determined, we believe it is likely to affect the biological function of the protein.

Our current knowledge about potential C1QTNF3-receptors and downstream signaling pathways is very limited. To date, Lysosomal-associated membrane protein 1, Lysosome membrane protein 2 (55) and Adiponectin receptor 2 (56) have been suggested as potential receptors for C1QTNF3 but the significance of these finding are yet largely unknown. However, several studies show that C1QTNF3 activates the MEK/ERK and the PI3K/Akt pathway and its beneficial metabolic and anti-inflammatory effects are thought to primarily depend on the PI3K/Akt pathway (26, 41, 57–59). In this study, we found that C1QTNF3 activates both these pathways in M2-type macrophages. However, the increase in Akt-phosphorylation was not seen until after 60 minutes of C1QTNF3-stimulation indicating that the PI3K/Akt pathway is not an immediate target of C1QTNF3 in this cell type. Moreover, Akt exists in three different isoforms and Akt1 and Akt2 can have opposing effects on macrophage polarization (60). The MEK/ERK1/2 pathway can also lead to opposing effects on macrophage polarization; it can be both a negative regulator of murine and human macrophage M2-type polarization (61) and an enhancer of M2-type polarization (62). While our experimental setting *in vitro* clearly reveals the pro-inflammatory action of C1QTNF3 as judged by the reduced proliferation of M0-type macrophages and the M1-polarizing effect, we also anticipate that the timing and the concentration of C1QTNF3, as well as the state and the type of the targeted macrophages, will determine whether C1QTNF3 primarily push M1- or M2-type polarization. For instance, the ERK1/2 activation that occur within minutes in response to growth factors leads to proliferation while the slower ERK1/2 activation in response to LPS leads to growth arrest and pro-inflammatory activation in bone marrow-derived macrophages (63). Notably, C1QTNF3 can also activate AMPK, the PKC signaling pathway and the cAMP/PKA pathway (64–68). Thus, we cannot rule out that additional mechanisms besides the ERK- and Akt-pathways are at play.

In conclusion, our data strengthen the notion that C1QTNF3 modulates signaling pathways in a cell- and (patho-)physiological-state-dependent manner as suggested by Petersen and colleagues (53). We propose that locally increased C1QTNF3 levels contribute to increased IWAT macrophage accumulation in response to a growing tumor. While this C1QTNF3-stimulated macrophage population appears not to be involved in tumor progression/rejection, its role in subcutaneous adipose tissue remodeling warrants further investigations.

## Data Availability Statement

The datasets for this study are included within the article and its supplementary material. RNA-sequencing data have been deposited in NCBI’s Gene Expression Omnibus (38) and are accessible through GEO Series accession number GSE201316.

## Ethics Statement

The animal study was reviewed and approved by The Regional Animal Ethics Committee in Gothenburg, Sweden.

## Author Contributions

IWA conceived the idea, supervised this work, interpreted data, made figures, and wrote the first draft of the manuscript. MV contributed conceptionally to this work, designed and performed experiments, interpreted data, made figures, wrote parts of the manuscript, and critically revised this manuscript. PM designed and performed experiments, interpreted data, made figures, and wrote parts of the manuscript. YWu, EP, YWa, and BC performed experiments and assisted in data interpretation. SC and AS gave valuable feedback to this work and assisted in data interpretation. All authors contributed to the article and approved the submitted version.

## Funding

This research was funded by the Swedish Research Council (2017-00792, 2017-01821, 2020-01463, 2013-7107, and 2020-01008); Swedish Cancer Society (19-0306, 18-0622, and 21-1674), Region Västra Götaland; the Swedish state under the agreement between the Swedish government and the county councils, the ALF-agreement (965065); Novo Nordisk Foundation (NNF19OC0056601); the Swedish Diabetes Foundation (DIA2019-419, DIA2016-127); the IngaBritt and Arne Lundberg Foundation and Wilhelm & Martina Lundgren Foundation.

## Conflict of Interest

AS is board member and declares stock ownership in Tulebovaasta, Iscaff Pharma and SiMSen Diagnostics.

The remaining authors declare that the research was conducted in the absence of any commercial or financial relationships that could be construed as a potential conflict of interest.

## Publisher’s Note

All claims expressed in this article are solely those of the authors and do not necessarily represent those of their affiliated organizations, or those of the publisher, the editors and the reviewers. Any product that may be evaluated in this article, or claim that may be made by its manufacturer, is not guaranteed or endorsed by the publisher.
