# Supplementary material for: C1QTNF3 is Upregulated During Subcutaneous Adipose Tissue Remodeling and Stimulates Macrophage Chemotaxis and M1-Like Polarization
Source: Front Immunol. 2022 Jun 2;13:914956. doi: 10.3389/fimmu.2022.914956 (PMC9202579; doi:10.3389/fimmu.2022.914956)
Supplement: Supplementary file 1 [file DataSheet_1.pdf]

frontiers

Supplementary Material

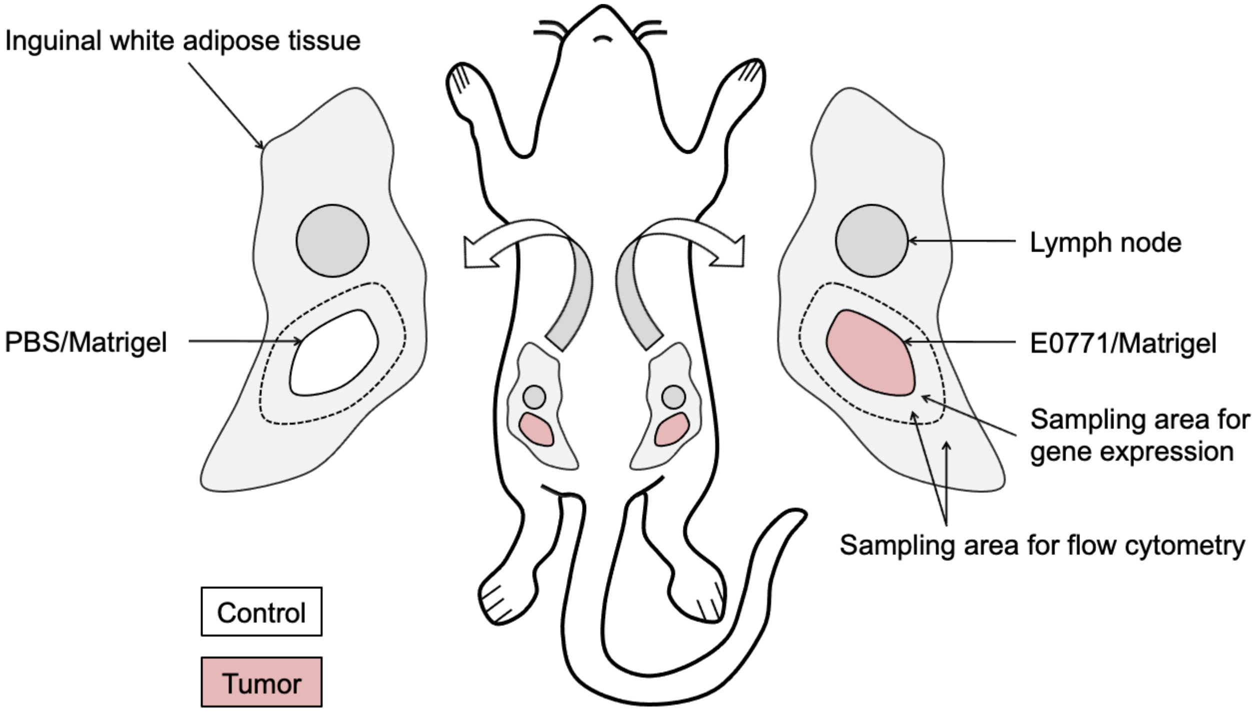

Supplementary Figure 1. Schematic overview of the E0771 breast cancer model and where cancer-associated and control adipose tissue were obtained for gene expression and flow cytometry analyses.

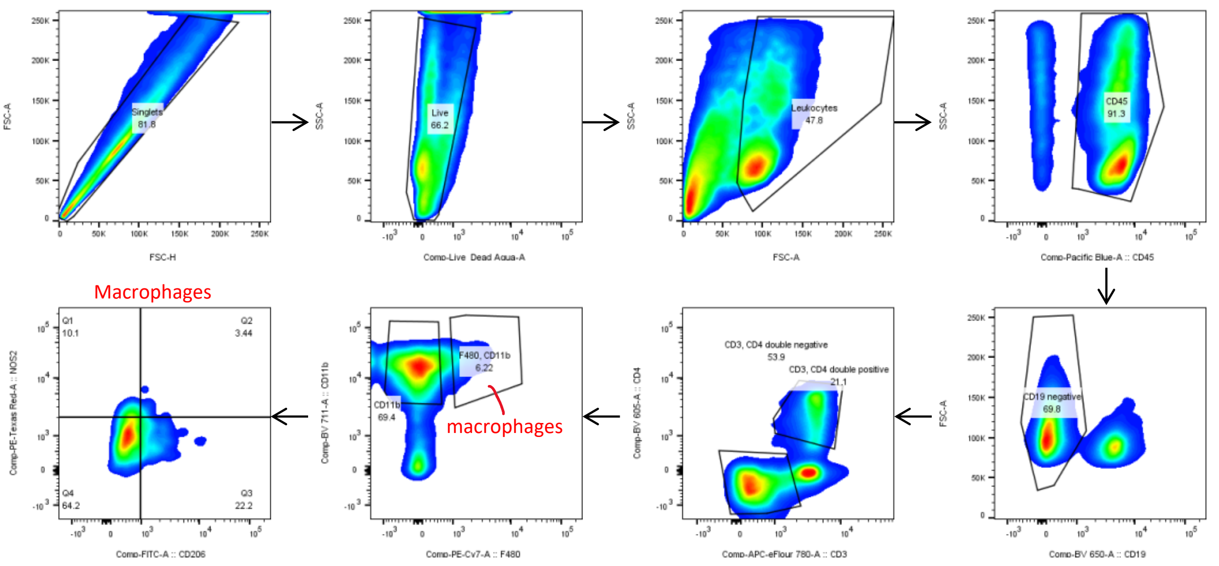

Supplementary Figure 2. Gating strategy for flow cytometry analysis of mouse tissues.

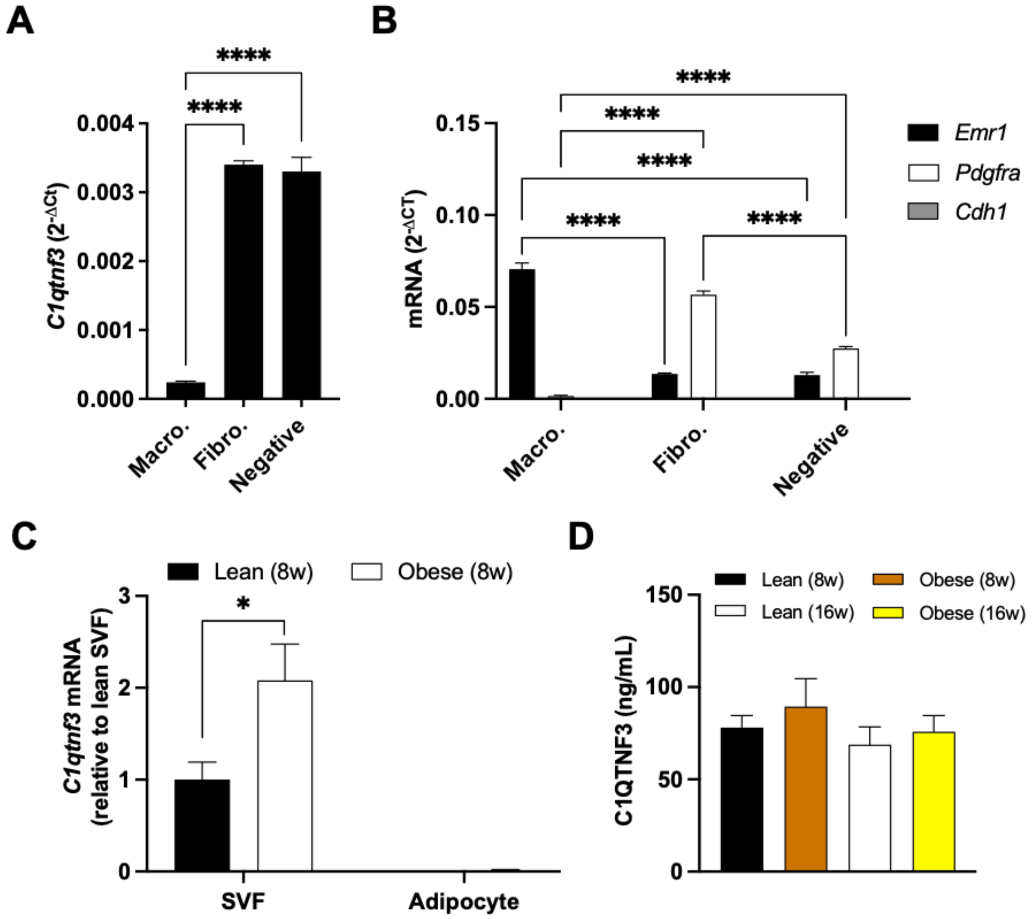

Supplementary Figure 3. (**A**) *C1qtnf3* expression in GWAT macrophages (F4/80$^+$), fibroblasts (F4/80$^-$CD45$^-$CD90.2$^+$) and in remaining F4/80$^-$CD45$^-$CD90.2$^-$ SVF cells (N = 3/group) and (**B**) analysis of cell population purity. (**C**) *C1qtnf3* expression in male IWAT SVF and adipocytes in response to 8-week (w) high fat diet (HFD) feeding (N = 7/group). (**D**) Serum C1QTNF3 levels in response to 8- and 16-week (w) HFD feeding in male mice (N = 5/group). * = p < 0.05, and **** = p < 0.0001 for the indicated comparisons.

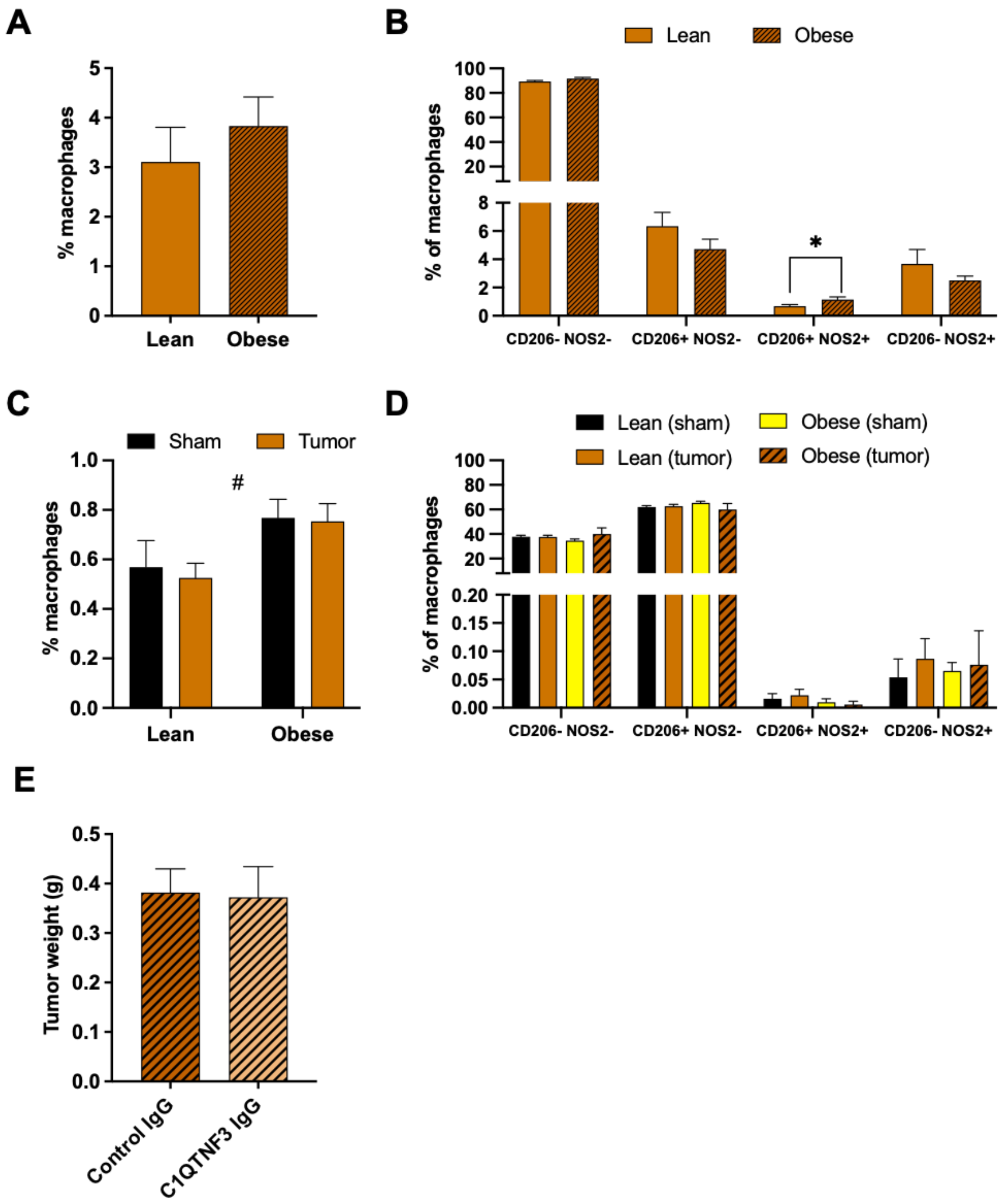

Supplementary Figure 4. (**A**) Total macrophages (% F4/80^{+}CD11b^{+} of viable cells) and (**B**) CD206^{-}NOS^{-}, CD206^{+}NOS2^{-} (M2-type), CD206^{+}NOS2^{+} (mixed M1-M2-type), CD206^{-}NOS2^{+} (M1-type) as % of total macrophages in tumors from lean and obese female mice (N =. 10/group). (**C**) Total macrophages and (**D**) their M1-M2 subset distribution in spleen from lean and HFD-induced obese female mice with or without orthotopic E0771 breast cancer (N = 4-7/group, # = $p<0.05$ for the effect of obesity in 2-way ANOVA). (**E**) Tumor weight in isotype control and C1QTNF3-IgG treated HFD-induced obese female mice (N = 6/group).

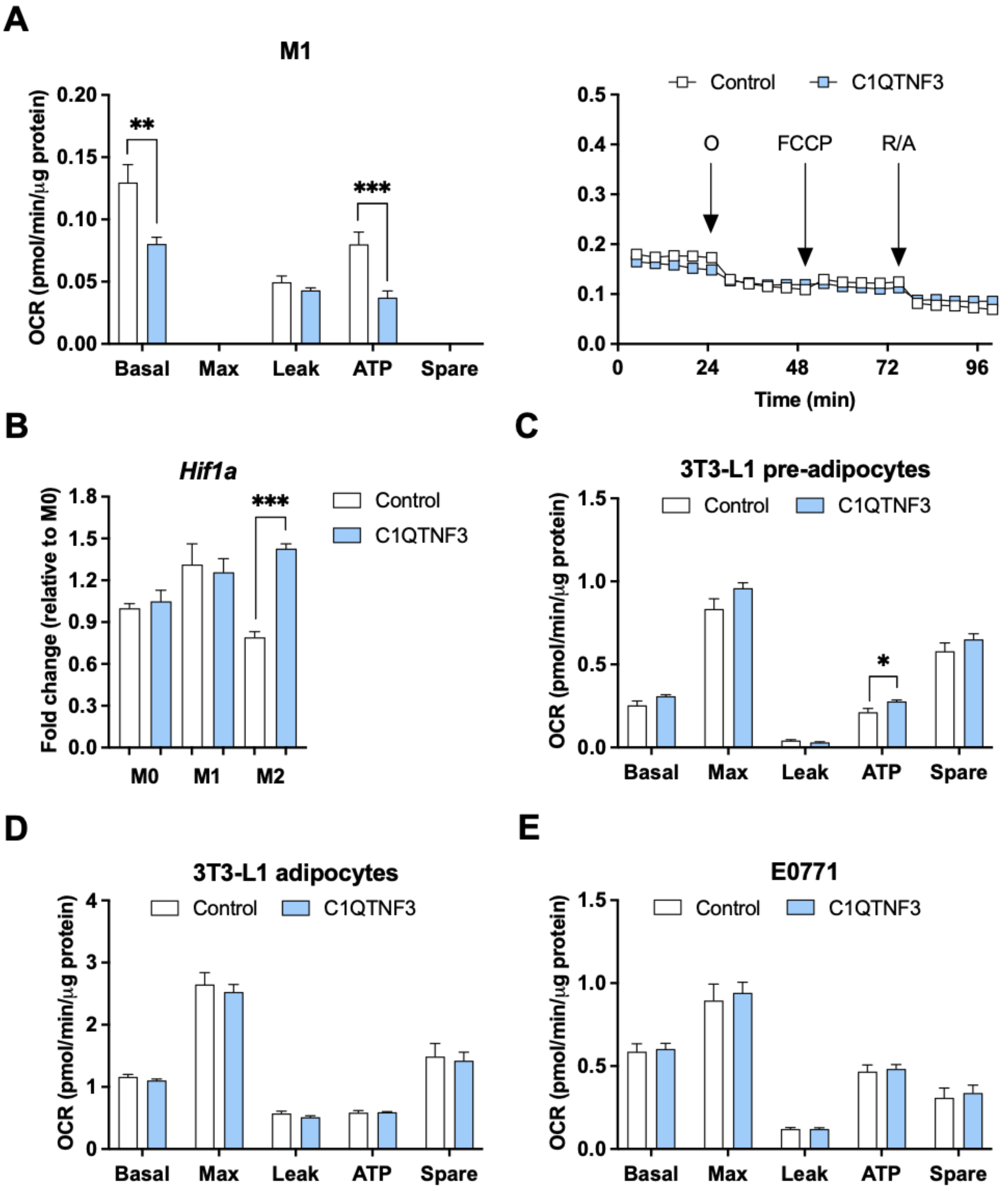

Supplementary Figure 5. Mitochondrial function, as determined by changes in basal respiration (Basal), maximal respiration (Max), proton leak-related respiration (Leak), ATP production-linked respiration (ATP) and spare respiratory capacity (Spare), was estimated from the oxygen consumption rate (OCR) of (**A**) cultured bone marrow derived M1-type macrophages in response to subsequent addition of oligomycin (O), FCCP, and rotenone (R)/antimycin A (A) as indicated in the right panels. Prior to these OCR measurements, the macrophages were treated with vehicle control solution or C1QTNF3 (1μg/ml) for 48 h along with LPS & IFNγ to induce M1- polarization. Data are presented as OCR normalized to total protein levels (N = 16/group). (**B**) *Hif1a* mRNA levels in cultured bone

marrow derived M0-, M1- and M2-type macrophages treated with or without C1QTNF3 (1µg/ml). Data is presented as fold change relative to the expression in the M0 control (N = 6/group). Mitochondrial function of cultured (**C**) 3T3-L1 pre-adipocytes, (**D**) 3T3-L1 adipocytes and (**E**) E0771 breast cancer cells treated with or without C1QTNF3 (1µg/ml) for 24h (N = 4-12/group). Data are presented as OCR normalized to total protein levels. * = $p < 0.05$, ** = $p < 0.01$, *** = $p < 0.001$ for the indicated comparison.
